# Immune Checkpoint Inhibitors‐Induced Endocrinopathies: Assessment, Management and Monitoring in a Comprehensive Cancer Centre

**DOI:** 10.1002/edm2.505

**Published:** 2024-06-26

**Authors:** Omayma Elshafie, Abir Bou Khalil, Bushra Salman, Abier Atabani, Hasan Al‐Sayegh

**Affiliations:** ^1^ Department of Endocrinology Sultan Qaboos Comprehensive Cancer Care and Research Center Muscat Oman; ^2^ Department of Pharmacy Sultan Qaboos Comprehensive Cancer Care and Research Center Muscat Oman; ^3^ Department of Medical Oncology Sultan Qaboos Comprehensive Cancer Care and Research Center Muscat Oman; ^4^ Department of Research Laboratories Sultan Qaboos Comprehensive Cancer Care and Research Center Muscat Oman

**Keywords:** checkpoint inhibitor–associated autoimmune diabetes (CIADM), endocrinopathies, hyperthyroidism, hypophysitis, hypothyroidism, immune checkpoint inhibitors, solid tumours, survival

## Abstract

**Objectives:**

To determine the incidence, presentation, frequency and management of immune checkpoint inhibitors (ICI)‐related endocrinopathies in a comprehensive cancer centre in Oman, particularly with programme death 1/programme death‐ligand 1 (PD‐1/PD‐L1) inhibitors.

**Background:**

A high number of patients treated with PD‐1/PD‐L1 inhibitors for the management of solid tumours developed endocrinopathies.

**Methods:**

This is a retrospective study of patients admitted to Sultan Qaboos Comprehensive Cancer Care and Research Centre (SQCCCRC) from August 2021 to December 2022. All adults diagnosed with solid cancers and have received at least one dose of ICIs were included. Patients with incomplete data were excluded from the analysis. Data regarding the ICI‐induced endocrinopathy were collected.

**Results:**

A total of 139 patients were included in the study of which 58% were females. The median age of the cohort was 56 years. The incidence of endocrine‐related adverse events was 28%. The mean time for the development of endocrine adverse events after treatment initiation was 4.1 ± 2.8 months. Of the patients who developed toxicity, 90% had hypothyroidism. Ten patients developed hyperthyroidism, two patients were diagnosed with secondary adrenal insufficiency/hypophysitis and one patient developed Type 1 diabetes mellitus (DM). Using univariable logistic regression weight and body mass index (BMI) significantly impacted the development of endocrine immune‐related adverse events (irAEs).

**Conclusions:**

This is the first study from the Sultanate of Oman to assess PD‐1/PDL‐1 ICI‐induced endocrinopathies. The most common endocrine adverse event is thyroid dysfunction, mainly hypothyroidism followed by hyperthyroidism. Hypophysitis, primary adrenal insufficiency and CIADM occur less frequently, but have a more significant effect on the patient's health. The treating physician should be aware of ICI‐induced endocrinopathies, screening and treatment. Furthermore, our study showed that patients with a higher BMI have a greater risk of developing irAES. Further studies are needed to establish the predictors of endocrine irAEs.

## Graphical abstract

The first retrospective study from the Sultanate of Oman to assess PD‐1/PDL‐1 ICI‐induced endocrinopathies. The most common endocrine adverse event is thyroid dysfunction. Less common but more serious adverse events include hypophysitis, primary adrenal insufficiency and Type I diabetes which physicians should be aware of.

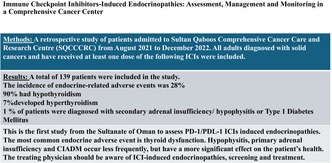

## 1 Introduction

The use of immune checkpoint inhibitors (ICIs) has transformed the treatment of cancer and is a standard of care in the treatment of many solid tumours [1]. There are two classes of ICIs currently licensed: those that inhibit cytotoxic T‐cell lymphocyte antigen‐4 (CTLA‐4) pathway, for example, ipilimumab and those that inhibit the programme death 1 receptor or ligand (PD‐1 or PD‐L1) signalling pathways, for example, atezolizumab, durvalumab, nivolumab and pembrolizumab. ICIs allow T cells to recognise and attack cancer cells by preventing the receptors and ligands from binding to each other [1]. One of the main challenges with the use of ICIs is that they frequently result in immune‐related adverse events (irAEs) affecting multiple organs, among which endocrine adverse events are common. The most common endocrine irAE in descending order are thyroid, pituitary, adrenal and beta cells of the pancreatic islets [2]. The pattern of endocrine irAEs differs between CTLA‐4 and PD‐1/PD‐L1 pathways. Hypothyroidism is more common with PD1/PD‐L1 inhibitors, whereas hypophysitis is more common with CTLA‐4 inhibitors [2, 3]. The combination of ICIs increases the risk of developing endocrinopathies [2, 3]. Unlike other irAEs, disruption of the endocrine system tends to be irreversible and leads to a need for lifelong hormone replacement [4].

The symptoms of endocrine dysfunction may be variable and nonspecific, posing a diagnostic challenge, which is further complicated by extensive use of corticosteroids and episodes of treatment‐induced immunosuppression [5]. Additionally, there is a considerable risk of hospitalisation and death if not recognised early [6]. Thus, clinicians need to be alert to the signs and symptoms of the likely endocrine consequences of ICIs, whereas endocrinologists need to be aware of the likely patterns of endocrine dysfunction.

In one study, PD‐1/PD‐L1 inhibition led to endocrine toxicity in 27.8% of cases in whom 75% developed thyroid dysfunction [7]. A large US‐based epidemiological study of more than 20,000 patients showed that the prevalence of endocrinopathies ranged between 10% and 18% for different types of ICIs [8]. To the best of our knowledge, no similar studies addressing the endocrine‐related AEs of ICIs were conducted in Oman.

Therefore, the aim of our study was to describe the incidence, presentation, frequency and management of ICI‐related endocrinopathies in our population, particularly with PD‐1/PD‐L1 inhibitors.

## 2 Methods

This is a single‐centre retrospective chart review of all patients who received ICIs at Sultan Qaboos Comprehensive Cancer Care and Research Centre (SQCCCRC) during the period from August 2021 to December 2022. All consecutive adolescent (13–18 years) and adult patients (>18 years of age) diagnosed with solid cancers and have received at least one dose of the following ICI (atezolizumab, durvalumab, nivolumab or pembrolizumab) were included. Included patients have received single agent ICI or in combination with chemotherapy and/or targeted therapy. Patients received ICIs in accordance with the NCCN guidelines for treatment indication, regimen, dosage and scheduling. Patients with incomplete data were excluded from predictive analysis.

Data collected included patient demographics (age, gender, height, weight, body mass index [BMI], family history of diabetes and thyroid disease, and Eastern Cooperative Oncology Group performance status [ECOG PS]), baseline laboratory data, tumour type and stage of the disease, the types and regimens of ICI used and number of ICI doses.

Additionally, laboratory data related to endocrine function and adverse events were collected including thyroid‐stimulating hormone (TSH), free thyroxine (T4), morning cortisol, adrenocorticotropic hormone (ACTH), glycated haemoglobin (HbA1c), fasting blood sugar, follicular stimulating hormone (FSH), oestradiol, luteinizing hormone (LH), testosterone level and electrolytes. The assessment done for suspected thyroid dysfunction, hypophysitis, adrenal insufficiency and Type 1 diabetes mellitus (DM) were also collected.

Moreover, data regarding the ICI‐induced endocrinopathies were collected including number of adverse events, onset of the adverse event and the cycle after which the adverse event developed. Endocrine adverse events were graded according to the National Cancer Institute Common Terminology Criteria for Adverse Events (NCI CTCAE) v5. Furthermore, symptoms, management and follow‐up of endocrinopathies were collected on the basis of chart documentation.

Descriptive analysis was performed using median and interquartile ranges (IQR) for the continuous variables, and frequency and proportions for the categorical variables. Univariable logistic regression was used to examine the effect of predictors on the study outcomes (endocrine adverse events). Odds ratios (OR) and 95% confidence intervals (CI) are reported. *p* values ≤ 0.05 were considered statistically significant. Analyses were performed using the Stata software (StataCorp. 2023. Stata Statistical Software: Release 18. College Station, TX: StataCorp LLC.).

## 3 Results

### 3.1 Patients' and Treatment Characteristics

A total of 139 patients were included in the study of which 58% were females. The median age of the cohort was 56 years (interquartile range: 42–67). Around 30% of the study population were elderly (65 years and above), and 12% were under 35 years. The median BMI was 24 kg/m^2^, with 20% of the group falling in the obese or morbidly obese range. The most diagnosed cancers were breast, lung and gastric cancer, in 33 (24%), 31 (22%) and 17 (12%) patients, respectively. Most patients (70%) had advanced Stage IV disease. Half of the patients had a low performance score (PS) of 0–1 at the initiation of ICI, whereas the other half had a PS of 2–3. One patient in the cohort has a PS of 4 upon treatment with ICI. Only 13 (17%) patients were prior or current smokers, whereas the remaining 113 patients did not have any smoking history.

**FIGURE 1:**
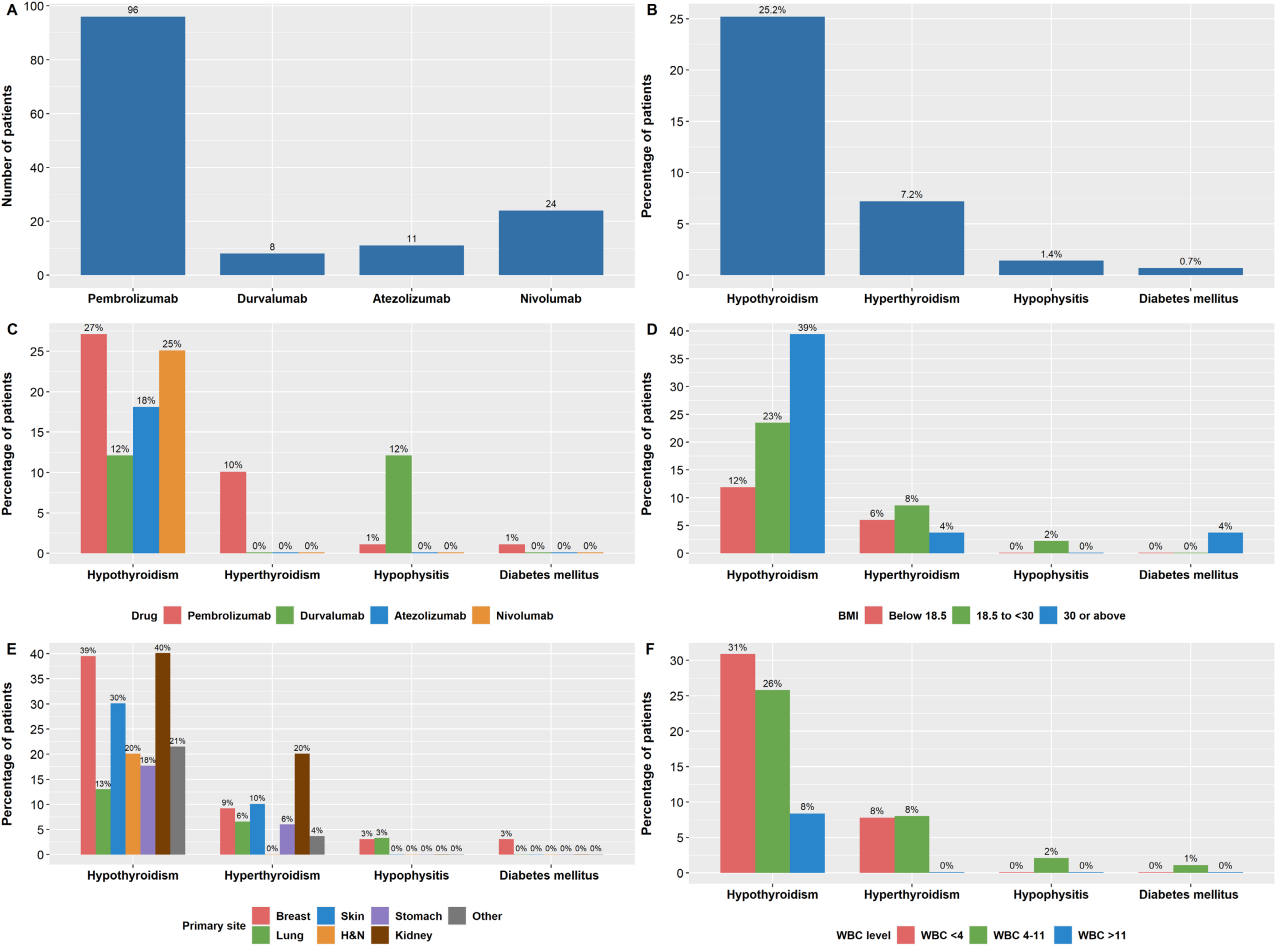
(A) Number of patients receiving ICI drugs. (B) Percentage of patients experiencing toxicity in the study population. (C) Percentage of patients experiencing toxicity by drug type. (D) Percentage of patients experiencing toxicity by BMI. (E) Percentage of patients experiencing toxicity by primary site. (F) Percentage of patients experiencing toxicity depending on baseline WBC count.

With regard to cancer treatment, most (69%) of the patients had received prior chemotherapy, around 50% had a prior surgery to the primary tumour or the metastases, and 36% received prior radiation. Ninety‐six patients (69%) in the cohort were treated with pembrolizumab, 24 (17%) patients received nivolumab, and 11 (8%) and 8 (6%) patients received atezolizumab and durvalumab, respectively (Figure 1A). Most patients received usual two‐ or three‐weekly doses rather than extended schedule four‐ or six‐weekly regimens. In the pembrolizumab cohort, only 10/96 received six‐weekly pembrolizumab. In the nivolumab cohort, 6/24 received the longer four‐weekly regimen. A similar practice was also followed with atezolizumab and durvalumab cohorts, where only one patient in each group received four‐weekly rather than two‐ or three‐weekly administrations. Patients were treated for a median of six cycles (range 1–33), translating into a treatment duration of <1 month to 40 months. All patient demographics and baseline characteristics and laboratory results are shown in Table 1.

**TABLE 1:** Baseline and laboratory characteristics.

Characteristic	*N* = 139
Gender (*n*, %)	
Male	59 (42)
Female	80 (58)
Age, years (median, IQR)	56 (42–67)
Age category (*n*, %)	
<35	16 (12)
35–64	81 (58)
>64	42 (30)
BMI, kg/m^2^ (median, IQR)	24 (20–29)
BMI category (*n*, %)	
<18.5	17 (12)
18.5–29.9	94 (68)
>30–39.9	24 (17)
>40	4 (3)
Primary tumour site (*n*, %)	
Breast	33 (24)
Lung	31 (22)
Stomach	17 (12)
Kidney	10 (7)
Head and neck	10 (7)
Skin	10 (7)
Others	28 (21)
Stage upon initiation of ICI (*n*, %)	
Stage II	16 (12)
Stage III	24 (18)
Stage IV	99 (70)
Metastatic site(s) (*n*, %)	
No metastasis	25 (18)
LN metastasis only	30 (21)
Bone or skin metastasis ± LNs	8 (6)
Visceral metastases	76 (55)
Performance status	
0	20 (14)
1	50 (36)
2	34 (24)
3	34 (24)
4	1 (1)
Smoking history (*n*, %)	
Yes	13 (17)
No	113 (81)
Missing	3 (2)
Comorbidity (*n*, %)	
Yes	74 (53.2%)
No	65 (46.8%)
Baseline laboratory tests (mean ± SD) [ref]	
Haemoglobin, g/dL	11 ± 2 [11–14.5]
WBC, 10^3^/μL	6.7 ± 3.9 [2.4–9.5]
Platelets, 10^3^/μL	340 ± 154 [150–450]
Creatinine, μmol/L	63 ± 23 [45–84]
ALT, U/L	21 ± 31 [0–33]
AST, U/L	23 ± 24 [0–32]
Bilirubin, μmol/L	6.8 ± 4.3 [0–17]
Baseline endocrine laboratory tests (mean ± SD) [ref]	
TSH, mIU/L	3.1 ± 7 [0.27–4.2]
T4, pmol/L	17 ± 3 [12.3–20.2]
Cortisol, nmol/L	339 ± 154 [133–537]
HbA1c, % (*N* = 54)	5.7 ± 1.4 [4.5–5.7]
Treatment history (missing = 1)	
Prior chemotherapy (*n*, %)	
Yes	111 (80)
No	27 (20)
Prior surgery (*n*, %)	
Yes	68 (49)
No	70 (51)
Prior radiation therapy (*n*, %)	
Yes	49 (36)
No	89 (64)
Type of ICI (*n*, %)	
Pembrolizumab	96 (69)
Nivolumab	24 (17)
Atezolizumab	11 (8)
Durvalumab	8 (6)
Number of doses of ICI received (median, IQR)	6 (4–10)

Abbreviations: ALT, alanine transaminase; AST, aspartate aminotransferase; BMI, body mass index; HbA1C, glycated haemoglobin; ICI, immune checkpoint inhibitor; IQR, interquartile range; LN, lymph node; ref, reference range; SD, standard deviation.

### 3.2 Incidence of Endocrine‐Related Adverse Events

The incidence of endocrine‐related adverse events in our study was around 28% (39/139) (Figure 1B). The mean time for the development of endocrine adverse events after treatment initiation was 4.1 ± 2.8 months (range 1–12 months). Of the patients who developed toxicity, 35 (90%) had hypothyroidism. Ten patients (7%) developed hyperthyroidism, two patients were diagnosed with secondary adrenal insufficiency/ hypophysitis, and one patient developed Type 1 DM. Isolated hypothyroidism was the only sign in most patients who developed toxicity (30 patients, 80%). Seven patients presented initially with hyperthyroidism, which then developed into hypothyroidism after 1–10 months of management. One patient who developed checkpoint inhibitor–associated autoimmune diabetes (CIADM), also developed hypothyroidism, which almost occurred concurrently, around 2 months after the initiation of the ICI pembrolizumab. Two patients developed secondary adrenal insufficiency due to hypophysitis (Figure 1C). The type and grade of endocrine adverse events are shown in Table 2.

**TABLE 2:** Pattern and grade of endocrine toxicities.

Endocrine toxicities	*N* = 139
Overall (*n*, %)	
Yes	39 (28)
No	100 (72)
Number of endocrine toxicities (*n*)	
1	30 (22)
2	8 (6)
3	1 (1)
Type of endocrine toxicity (*n*, %)	
Hypothyroidism	35 (25)
Grade I	14
Grade II	14
Grade III	7
Hyperthyroidism (Grade I)	10 (7)
Secondary adrenal insufficiency due to hypophysitis (Grade II)	2 (1)
Diabetes mellitus (Type 1) (Grade III)	1 (1)

### 3.3 Predictors of Endocrine‐Related Adverse Events

In the univariable logistic regression, age and sex were not associated with endocrine irAEs in our study. On the other hand, both weight and BMI significantly impacted the development of endocrine irAEs, although the magnitude of effect was small. The mean weight in patients who developed toxicity was 70 kg compared with 61 kg in patients who did not (OR = 1.023, 95% CI = 1.003–1.044, *p* value = 0.027). Similarly, the mean BMI in those with toxicity was 28 kg/m^2^ compared to 24 kg/m^2^ in patients without endocrine irAEs (OR = 1.069, 95% CI = 1.016–1.124, *p* value = 0.006). Patients diagnosed with genitourinary cancers had a significantly higher risk of developing endocrine irAEs (OR = 4.043, 95% CI = 1.301–12.562, *p* value = 0.016). The presence of any comorbidity versus no comorbidity was a significant negative predictor of toxicity (OR = 0.434, 95% CI = 0.203–0.927, *p* value = 0.031). Conversely, a higher disease stage (namely Stage IV) did not predict toxicity in our cohort. Likewise, prior history of treatment including surgery, radiation or chemotherapy was not associated with toxicity, nor was the current ICI type. Moreover, PS and smoking history were not found to be significantly related to the development of ICI toxicity. Of the laboratory tests, only WBC and AST had a significant association with endocrine irAEs. A lower WBC was associated with a higher risk of developing ICI‐related endocrinopathy (OR = 0.832, 95% CI = 0.711–0.973, *p* value = 0.021). A similar pattern of association was also seen with AST (OR = 0.876, 95% CI = 0.793–0.968, *p* value = 0.009). With regard to the treatment regimen, a higher number of received doses predicted a higher toxicity level (OR = 1.063, 95% CI = 1.000–1.131, *p* value = 0.042). The predictive value of several baseline and laboratory characteristics is shown in Table 3. Figure 1D–F show the percentage of patients experiencing toxicity by BMI, tumour site and WBC level.

**TABLE 3:** Predictors of endocrine‐related toxicity in the univariable regression model.

Risk factor	OR (95% CI)	*p*
Gender (female vs. male)	1.703 (0.786–3.692)	0.177
Age (year)	0.998 (0.973–1.024)	0.895
Weight (kg)	1.023 (1.003–1.044)	0.027*
BMI (kg/m^2^)	1.069 (1.016–1.124)	0.006*
GU programme (vs. other programmes)	4.043 (1.301–12.562)	0.016*
Stage IV disease (vs. stage II–III)	0.627 (0.284–1.386)	0.249
Comorbidity (vs. no comorbidity)	0.434 (0.203–0.927)	0.031*
Baseline Hb (g/dL)	0.966 (0.760–1.229)	0.780
Baseline platelets (10^3^/μL)	1.001 (0.999–1.004)	0.336
Baseline WBC (10^3^/μL)	0.832 (0.711–0.973)	0.021*
Smoking (vs. no smoking)	0.489 (0.155–1.546)	0.223
PS ≥ 2 (vs. PS < 2)	1.688 (0.798–3.571)	0.171
Baseline creatinine (μmol/L)	1.001 (0.990–1.022)	0.499
Baseline AST (U/L)	0.876 (0.793–0.968)	0.009*
Baseline TSH (mIU/L)	0.964 (0.857–1.083)	0.536
Baseline cortisol (nmol/L)	1.000 (0.996–1.001)	0.394
Baseline HbA1c (%)	0.696 (0.417–1.162)	0.167
Prior chemotherapy (vs. no prior chemotherapy)	0.707 (1.300–1.665)	0.428
Prior surgery (vs. prior surgery)	1.790 (0.815–3.929)	0.147
Prior radiation (vs. prior radiation)	0.808 (0.351–1.859)	0.616
ICI type		
Nivolumab vs. pembrolizumab	0.733 (0.246–1.948)	0.551
Atezolizumab vs. pembrolizumab	0.489 (0.072–2.043)	0.378
Durvalumab vs. pembrolizumab	0.314 (0.016–1.878)	0.289
Dosing schedule (2‐ or 3‐weekly vs. 4‐ or 6‐weekly)	1.107 (0.734–1.670)	0.629
No. of doses received	1.063 (1.000–1.131)	0.042*

Abbreviations: ALT, alanine transaminase; AST, aspartate aminotransferase; BMI, body mass index; GU, genitourinary; Hb, haemoglobin; HbA1C, glycated haemoglobin; ICI, immune checkpoint inhibitor; PS, performance status; TS, thyroid‐stimulating hormone; WBC, white blood count.

* *p* < 0.05 and hence significant.

We constructed a multivariable logistic regression model including the significant predictors in the univariable analyses including BMI, WBC, AST, GU programme, comorbidity and number of doses. We did not include weight in the multivariable model because it was strongly correlated with the BMI. Including both weight and BMI in the same model would introduce collinearity and violate the logistic regression assumptions. All variables in the multivariable model remained significant except WBC and number of doses. We interpret the results of the multivariable model with caution due to the low number of outcomes (endocrine adverse events) relative to the number of predictors.

### 3.4 Management of Endocrine‐Related Adverse Events

ICI was withheld due to endocrine toxicity only in 4 out of 39 cases (10%). Two patients with severe hypothyroidism (Grade 3), one patient with hypophysitis and one patient with CIADM withheld treatment due to irAEs. All patients with hypothyroidism started on levothyroxine with the mean dose being 100 μg (range 25–150 μg). The patients with symptomatic hyperthyroidism were started on the beta blocker propranolol, and the rest were monitored until recovery or development of hypothyroidism. Two patients with hypophysitis were started on hydrocortisone. One patient required stress dose hydrocortisone then tapered to maintenance dose 20 mg AM and 10 mg PM. The second patient was initiated on maintenance hydrocortisone. The patient who developed CIADM started on insulin mixtard 30 which was adjusted to achieve blood sugar control.

## 4 Discussion

Endocrinopathies with immune checkpoint inhibitor therapy presents a unique clinical challenge for the oncologists and internists who faces the need to identify endocrine dysfunction in patients on ICIs with often nonspecific symptoms or complex abnormal laboratory findings. Our study is the first study in Oman to describe the incidence, presentation, frequency and management of ICI‐related endocrinopathies.

### 4.1 Thyroid Dysfunction

Among endocrinopathies, thyroid dysfunction mainly primary hypothyroidism is the most common irAE. In our study, 25.2% of patients developed primary hypothyroidism of which 23% (seven patients) initially had hyperthyroidism. Hypothyroidism occurred in most cases in the first 3 months of treatment, but some patients developed hypothyroidism up to 10 months posttreatment. Early studies showed that the rate of hypothyroidism post‐PD1 inhibition was around 5% but this increased to up to 20% in later studies [9]. The incidence of hypothyroidism in our study was slightly higher than previous studies done outside the Arab World where most of our patients who developed hypothyroidism were on pembrolizumab. On the other hand, in two tertiary centres in the UAE the prevalence of hypothyroidism was 27%, which is similar to the incidence in our centre [10]. This might point out that patients in GCC (Gulf Cooperation Council) are at a higher risk of developing hypothyroidism. Hypothyroidism was permanent and required lifelong levothyroxine replacement.

On the other hand, hyperthyroidism was the second most common irAE in 7% of patients and was detected in the first month of treatment in most cases. All patients had painless transient thyrotoxicosis of which 70% became permanently hypothyroid. Most patients were asymptomatic during hyperthyroidism and discovered due to screening thyroid function tests while on ICIs. Our patients' thyroid glands were not enlarged and there was no exophthalmos to suspect Grave's disease [11]. Only a few patients required betablocker for symptomatic tachycardia. Similar to our findings, the study by Sakakida et al. [12] showed that more than 50% of the patients became hypothyroid post‐transient thyrotoxicosis but the detection of hyperthyroidism was seen in later stage as majority of patients were asymptomatic.

Several studies have found an association between thyroid autoantibodies (thyroyperoxidase or thyroglobulin) and risk of ICI‐induced thyroid dysfunction, and in one study approximately all patients with thyroid autoantibodies developed hypothyroidism [11, 13]. In our centre, thyroperoxidase antibody tests are done at referral laboratories and hence were not performed. However, the exact mechanism underlying immunotherapy‐induced thyroid dysfunction is still unclear, and expression PD‐L1 and PD‐L2 in the thyroid tissue may play a role [14].

As thyroid dysfunction is the most common irAE screening with TSH and free T4 remains key for early detection and treatment of patients. Screening should be performed at baseline then every 4–6 weeks during treatment for early detection and treatment [1, 4]. Screening every 3–6 months posttreatment with TSH and free T4 should be considered as patients may develop thyroid dysfunction posttreatment discontinuation.

Thyroid dysfunction as an irAEs may have positive effect on cancer. In one study, thyroiditis post‐ICI improved overall survival in patients especially those with lung cancer. The greater effect in lung cancer may be attributed to shared developmental origin of thyroid and lung epithelia [15]. On the other hand, a systematic review and meta‐analysis found low‐strength correlations between specific irAE rates and overall survival in several cancer types [16]. In a recent systematic review and meta‐analysis evidence suggested that the development of thyroid‐related adverse events is associated with antitumour effects of ICIs and may be a useful surrogate marker for clinical response [17]. In addition, it is associated with improved survival in some cancers possibly due to the presence of shared antigens between certain cancers and the thyroid [17]. This implies that further investigation for longer periods may be needed to arrive at a stronger conclusion.

### 4.2 Hypophysitis

Hypophysitis is a rare irAE in patients on PD‐1/PDL‐1 inhibitors occurring in around 1% of patients and at median of 4.5 cycles [4, 18]. Unlike patients on CTLA‐4 inhibitors, patients on PD‐1/PDL‐1 inhibitors may not report headache and their MRI may be normal. In addition, some patients on PD‐1/PDL‐1 may have isolated secondary adrenal insufficiency and be asymptomatic [11]. In our study, two patients developed hypophysitis (1.4%). The first patient developed fatigue without any headache or vision changes after the second cycle of pembrolizumab 400 mg every 6 weeks. He had an MRI brain without signs of metastasis or pituitary enlargement. He was started on hydrocortisone replacement therapy. The second patient developed secondary adrenal insufficiency along with hypogonadism 6 months post‐ICI. The patient presented with sepsis and inability to withdraw from inotropes leading to assessment of pituitary adrenal axis. The patient's ICI was withheld initially and started on stress doses of steroid which was tapered later to replacement dose. To note that patient developed subclinical primary hypothyroidism at 7 months of treatment and 1 month after adrenal insufficiency. The patient's MRI pituitary was normal. Our study is similar to previous studies where hypophysitis in patients on PD‐1/PDL‐1 inhibitors occur rarely. One of our patients had mild symptoms as described in previous studies, whereas the other patient had septic shock and required ICU admission due to secondary adrenal insufficiency. Therefore, it is always important to screen regularly for adrenal insufficiency by cortisol morning test as patients may have an indolent presentation and may not present any symptoms of headache, change in vision, weight loss, nausea, vomiting, hypotension, hypoglycaemia and weight loss. In patients with confirmed secondary adrenal insufficiency, screen for other hormonal disturbances mainly TSH, free T4 and electrolytes is recommended. Also, to consider evaluating LH and testosterone in males with changes in libido and FSH, and oestrogen in premenopausal females with menstrual changes. Additional considerations include MRI brain/sella with new hormonal deficiencies and particularly those with multiple endocrine abnormalities, new severe headaches or complaints of vision changes [1].

### 4.3 Primary Adrenal Insufficiency

Primary adrenal insufficiency rarely occurs in patients on ICIs and usually develops after several months of treatment [4, 14]. The range for developing adrenal insufficiency varies widely from weeks to more than 12 months [4] The symptoms may be nonspecific and include abdominal pain, nausea, weight loss, fatigue, anorexia, hypotension and hypoglycaemia [14]. Its prevalence may be underestimated due to concomitant treatment with glucocorticoids or the coexistence of secondary adrenal insufficiency [11]. Adrenal crisis has been reported in some cases with hyponatremia being the main sign [19]. In our study, none of the patients developed primary adrenal insufficiency. This may be due to concomitant treatment with glucocorticoids, failure to screen all patients with cortisol morning test regularly, the low incidence of primary adrenal insufficiency with ICI therapy or irAE occurring after finishing treatment. Therefore, it is important to screen patients with AM cortisol and if suspecting primary adrenal insufficiency to continue workup with ACTH, electrolytes, renin and aldosterone every 1–2 cycles followed by screening cortisol every 3–6 months once treatment is stopped to detect adrenal insufficiency and prevent adrenal crisis.

### 4.4 Checkpoint Inhibitor–Associated Autoimmune Diabetes (CIADM)

CIADM is a rare and distinct form of autoimmune diabetes occurring in 0.1%–1.4% of patients [11, 20]. CIADM is characterised by abrupt permanent beta cell failure which occurs at around 12 weeks post‐ICI initiation and up to 63 weeks from treatment. The patients usually develop acute onset hyperglycaemia and have low C‐peptide at onset of symptoms [11, 20]. As compared to patients with Type 1DM, patients with CIADM have less autoantibodies (40% vs. 90%), greater risk to develop diabetic ketoacidosis (DKA) at diagnosis (69.7% vs. 39%), no honeymoon effect and increase in pancreatic enzymes [20]. One patient out of 139 developed CIADM in our study (0.7%). The patient was not diabetic prior to treatment initiation with an HBA1c of 5.4%. After 3 months of treatment her blood sugar increased significantly reaching 30 mmol/L and the patient was started on insulin. Our patient had another irAE prior to CIADM which is hypothyroidism a month prior to diabetes development. Anti‐GAD and C‐peptide done 3 months post diagnosis of diabetes showed positive anti‐GAD of 45 IU/mL (<10), low C‐peptide 0.61 ng/mL (0.81–3.85). Pancreatic enzymes were not performed at the time of diagnosis. The patient's HBA1c was still in the prediabetic range at 6 mmol/L when she was diagnosed with CIADM. The proposed diagnostic criteria for CIADM are hyperglycaemia (blood sugar >11 mmol/L or Hba1c > or 6.5%) and insulin resistance (low C‐peptide or DKA) at diagnosis and testing can be repeated at 1 month in those with clinical concern for CIADM but not meeting criteria at presentation [20]. Therefore, it is critical to monitor the symptoms of new or worsening diabetes (polyuria and polydipsia) in patients on ICI. A baseline glucose with each cycle then in follow‐up visits for at least 6 months. If CIADM is suspected then additional laboratories should be sent including serum/urine ketones, electrolytes (for anion gap), C‐peptide and Type 1DM antibodies (anti‐GAD or anti‐islet cell antibodies) as patients have high risk to develop DKA and need treatment with insulin rather than oral antidiabetic medications [1].

### 4.5 Predictors of Endocrine‐Related Adverse Event

Deciphering the mechanisms driving the immune‐related adverse will help in early diagnosis, treatment and even prevention of such side effects. In our study, a higher BMI increased the risk of developing endocrine‐related adverse events. Similarly in one study and in a meta‐analysis, overweight and obesity appeared to be correlated with an increased incidence of any‐grade irAEs [21, 22]. The findings may suggest that patients with obesity be monitored for AEs more closely than lean patients.

Different cancer types treated with ICI have different risks to develop irAEs. In our study, patients with genitourinary cancer had more endocrine‐related irAEs compared with other tumours. However, the risk of chance finding cannot be ruled out due to the small sample size of patients with genitourinary cancers in our study. Additionally, this diagnosis, occurring mostly in advanced age, might have correlations with other variables such as comorbidities. Other systematic reviews have also shown that different tumour histologies have a different irAE profile when treated with PD‐1 inhibitors [23]. This could be attributed to the tumour microenvironment, immune infiltrate and adaptive immune response [23]. Further and larger studies are needed to assess the risk of developing endocrinopathies in different cancers due to PD‐1 inhibitors.

In our study, unexpectedly lower WBC at baseline was associated with a higher risk of developing ICI‐related endocrinopathy. Several studies have explored the effect of blood cell count and its changes during treatment on irAEs. A study showed that increased WBC and decreased lymphocyte count during treatment increased risk of irAEs as lymphopaenia may suggest impaired cell immunity [24]. Another study showed that increased baseline eosinophil count might suggest increased risk for irAEs [24]. More studies are needed to explore the changes in blood count and their impact on irAEs, and especially endocrinopathies.

### 4.6 Study Limitations

Our study possesses several limitations that necessitate acknowledgment. These include the retrospective design of the study leading to incomplete data collection and incomplete follow‐up. In addition, the absence of a standardised testing protocol for assessing endocrine adverse effects where not all patients are initially screened and regularly followed up for endocrine adverse effects. Hence irAEs may not be detected or detected at a later stage. The possibility of checking thyroid autoantibodies and assessing its relation to developing in irAEs is needed in our centre. Furthermore, our study is from a single centre with a relatively small sample size. Hence, the generalisability of our results may be hindered.

## 5 Conclusion

This is the first study from Sultanate Oman to assess PD‐1/PDL‐1 ICIs induced endocrinopathies. The most common endocrine adverse event is thyroid dysfunction, mainly hypothyroidism followed by hyperthyroidism. Most cases do not require withholding ICI and hypothyroid patients need lifelong replacement. Hypophysitis, primary adrenal insufficiency and CIADM occur less frequently, but have a more significant effect on the patient's health. The treating physician should always have high suspicion regarding hypophysitis, primary adrenal insufficiency and CIADM as symptoms may be nonspecific and will require lifelong treatment. In addition, screening for endocrinopathies in patients on ICI and after withholding ICI is essential to detect early and treat the endocrinopathies. An endocrine irAE screening protocol at initiation and follow‐up may be helpful for better detection and faster treatment of patients. Furthermore, our study showed that patients with a higher BMI have a greater risk of developing irAES and a lower WBC and AST are associated with a higher risk of developing ICI‐related endocrinopathy. ARAB The risk of developing irAES may also be related to the cancer itself. Further studies are needed to well establish predictors of endocrine‐related adverse events.

## 6 Author Contributions

**Omayma Elshafie:** management of patient, data collection and article writing. **Abir Bou Khalil:** management of patient, data collection and article writing. **Bushra Salman:** data analysis and writing of part of results. **Abeir Atabani:** data collection. **Hasan Al‐Sayegh:** statistical analysis and part of results writing.

## Conflicts of Interest

The authors declare no conflicts of interest.

## Data Availability

The data that support the findings of this study are available from the corresponding author upon reasonable request.

## References

[1] B. J. Schneider , J. Naidoo , B. D. Santomasso , et al., “Management of Immune‐Related Adverse Events in Patients Treated With Immune Checkpoint Inhibitor Therapy: ASCO Guideline Update,” Journal of Clinical Oncology 39, no. 36 (2021): 4073–4126, 10.1200/jco.21.01440. 34724392

[2] J. J. Wright , A. C. Powers , and D. B. Johnson , “Endocrine Toxicities of Immune Checkpoint Inhibitors,” Nature Reviews Endocrinology 17, no. 7 (2021): 389–399, 10.1038/s41574-021-00484-3. PMC8769055 33875857

[3] J. de Filette , C. Andreescu , F. Cools , B. Bravenboer , and B. Velkeniers , “A Systematic Review and Meta‐Analysis of Endocrine‐Related Adverse Events Associated With Immune Checkpoint Inhibitors,” Hormone and Metabolic Research 51, no. 3 (2019): 145–156, 10.1055/a-0843-3366. 30861560

[4] J. Haanen , M. Obeid , L. Spain , et al., “Management of Toxicities From Immunotherapy: ESMO Clinical Practice Guideline for Diagnosis, Treatment and Follow‐Up,” Annals of Oncology 33 (2022): 1217–1238, 10.1016/j.annonc.2022.10.001. 36270461

[5] R. Hattersley , M. Nana , and A. J. Lansdown , “Endocrine Complications of Immunotherapies: A Review,” Clinical Medicine 21, no. 2 (2021): e212–e222, 10.7861/clinmed.2020-0827. 33762389 PMC8002767

[6] E. Nogueira , T. Newsom‐Davis , and D. L. Morganstein , “Immunotherapy‐Induced Endocrinopathies: Assessment, Management and Monitoring,” Therapeutic Advances in Endocrinology and Metabolism 10 (2019): 204201881989618, 10.1177/2042018819896182. PMC6933543 31903179

[7] R. Rubino , A. Marini , G. Roviello , et al., “Endocrine‐Related Adverse Events in a Large Series of Cancer Patients Treated With Anti‐PD1 Therapy,” Endocrine 74, no. 1 (2021): 172–179, 10.1007/s12020-021-02750-w. 34036513 PMC8440282

[8] D. Elantably , A. R. Al Armashi , F. Hammad , and A. Alkrekshi , “Immune Checkpoint Inhibitor‐Related Endocrinopathies: A Nationwide Population‐Based Study,” Journal of Clinical Oncology 40, no. 16_suppl (2022): 2653, 10.1200/jco.2022.40.16_suppl.2653.

[9] M. Girotra , A. Hansen , A. Farooki , et al., “The Current Understanding of the Endocrine Effects From Immune Checkpoint Inhibitors and Recommendations for Management,” JNCI Cancer Spectrum 2, no. 3 (2018): pky021, 10.1093/jncics/pky021. 30057972 PMC6054022

[10] M. Omara , E. Abdelgadir , F. Khan , et al., “Incidence of Immune Related Adverse Events in Patients Treated With Immune Checkpoint Inhibitors, Case Series From Two Tertiary Care Centeers in Dubai, UAE,” Tumori Journal 106, no. 1 Suppl (2020): 25, 10.1177/0300891620914156.31456509

[11] K. C. J. Yuen , S. L. Samson , I. Bancos , et al., “American Association of Clinical Endocrinology Disease State Clinical Review: Evaluation and Management of Immune Checkpoint Inhibitor‐Mediated Endocrinopathies: A Practical Case‐Based Clinical Approach,” Endocrine Practice 28, no. 7 (2022): 719–731, 10.1016/j.eprac.2022.04.010. 35477029

[12] T. Sakakida , T. Ishikawa , J. Uchino , et al., “Clinical Features of Immune‐Related Thyroid Dysfunction and Its Association With Outcomes in Patients With Advanced Malignancies Treated by PD‐1 Blockade,” Oncology Letters 18 (2019): 2140–2147, 10.3892/ol.2019.10466. 31423288 PMC6607381

[13] A. Labadzhyan , K. Wentzel , O. Hamid , et al., “Endocrine Autoantibodies Determine Immune Checkpoint Inhibitor‐Induced Endocrinopathy: A Prospective Study,” The Journal of Clinical Endocrinology & Metabolism 107, no. 7 (2022): 1976–1982, 10.1210/clinem/dgac161. 35303106 PMC9202695

[14] N. Okura , M. Asano , J. Uchino , et al., “Endocrinopathies Associated With Immune Checkpoint Inhibitor Cancer Treatment: A Review,” Journal of Clinical Medicine 9, no. 7 (2020): 2033, 10.3390/jcm9072033. 32610470 PMC7409155

[15] S. Street , D. Chute , I. Strohbehn , et al., “The Positive Effect of Immune Checkpoint Inhibitor‐Induced Thyroiditis on Overall Survival Accounting for Immortal Time Bias: A Retrospective Cohort Study of 6596 Patients,” Annals of Oncology 32, no. 8 (2021): 1050–1051, 10.1016/j.annonc.2021.05.357. 34020034 PMC8814352

[16] V. Amoroso , F. Gallo , A. Alberti , et al., “Immune‐Related Adverse Events as Potential Surrogates of Immune Checkpoint Inhibitors' Efficacy: A Systematic Review and Meta‐Analysis of Randomized Studies,” ESMO Open 8, no. 2 (2023): 100787, 10.1016/j.esmoop.2023.100787. 36842300 PMC9984799

[17] Y.‐M. M. Cheung , W. Wang , B. McGregor , and O.‐P. R. Hamnvik , “Associations Between Immune‐Related Thyroid Dysfunction and Efficacy of Immune Checkpoint Inhibitors: A Systematic Review and Meta‐Analysis,” Cancer Immunology, Immunotherapy 71, no. 8 (2022): 1795–1812, 10.1007/s00262-021-03128-7. 35022907 PMC9276851

[18] J. Johnson , W. Goldner , D. Abdallah , F. Qiu , A. K. Ganti , and A. Kotwal , “Hypophysitis and Secondary Adrenal Insufficiency From Immune Checkpoint Inhibitors: Diagnostic Challenges and Link With Survival,” Journal of the National Comprehensive Cancer Network 21, no. 3 (2023): 281–287, 10.6004/jnccn.2022.7098. 36828029

[19] H. Trainer , P. Hulse , C. E. Higham , P. Trainer , and P. Lorigan , “Hyponatraemia Secondary to Nivolumab‐Induced Primary Adrenal Failure,” Endocrinology, Diabetes & Metabolism Case Reports 2016 (2016a): 16‐0108, 10.1530/edm-16-0108. PMC5097140 27857838

[20] L. Wu , V. Tsang , A. M. Menzies , et al., “Risk Factors and Characteristics of Checkpoint Inhibitor–Associated Autoimmune Diabetes Mellitus (CIADM): A Systematic Review and Delineation From Type 1 Diabetes,” Diabetes Care 46, no. 6 (2023): 1292–1299, 10.2337/dc22-2202. 37220262

[21] J. L. McQuade , H. Hammers , H. Furberg , et al., “Association of Body Mass Index With the Safety Profile of Nivolumab With or Without Ipilimumab,” JAMA Oncology 9, no. 1 (2023): 102–111, 10.1001/jamaoncol.2022.5409. 36480191 PMC9857666

[22] Y. Guzman‐Prado , J. Ben Shimol , and O. Samson , “Body Mass Index and Immune‐Related Adverse Events in Patients on Immune Checkpoint Inhibitor Therapies: A Systematic Review and Meta‐Analysis,” Cancer Immunology, Immunotherapy 70, no. 1 (2020): 89–100, 10.1007/s00262-020-02663-z. 32648164 PMC10991299

[23] L. Khoja , D. Day , T. Wei‐Wu Chen , L. L. Siu , and A. R. Hansen , “Tumour‐ and Class‐Specific Patterns of Immune‐Related Adverse Events of Immune Checkpoint Inhibitors: A Systematic Review,” Annals of Oncology 28, no. 10 (2017): 2377–2385, 10.1093/annonc/mdx286. 28945858

[24] X. Liu , Y. Shi , D. Zhang , et al., “Risk Factors for Immune‐Related Adverse Events: What Have We Learned and What Lies Ahead?” Biomarker Research 9, no. 1 (2021): 79, 10.1186/s40364-021-00314-8. 34732257 PMC8565046

